# *In Vitro* Maturation of Cumulus-Oocyte Complexes for Efficient Isolation of Oocytes from Outbred Deer Mice

**DOI:** 10.1371/journal.pone.0056158

**Published:** 2013-02-14

**Authors:** Jung Kyu Choi, Xiaoming He

**Affiliations:** 1 Department of Biomedical Engineering, The Ohio State University, Columbus, Ohio, United States of America; 2 Davis Heart and Lung Research Institute, The Ohio State University, Columbus, Ohio, United States of America; 3 James Comprehensive Cancer Center, The Ohio State University, Columbus, Ohio, United States of America; Institut Jacques Monod - UMR 7592 CNRS - Université Paris Diderot, France

## Abstract

**Background:**

The outbred (as with humans) deer mice have been a useful animal model of research on human behavior and biology including that of the reproductive system. One of the major challenges in using this species is that the yield of oocyte isolation via superovulation is dismal according to the literature to date less than ∼5 oocytes per animal can be obtained so far.

**Objective:**

The goal of this study is to improve the yield of oocyte isolation from outbred deer mice close to that of most laboratory mice by *in vitro* maturation (IVM) of cumulus-oocyte complexes (COCs).

**Methods:**

Oocytes were isolated by both superovulation and IVM. For the latter, COCs were obtained by follicular puncture of antral follicles in both the surface and inner cortical layers of ovaries. Immature oocytes in the COCs were then cultured *in vitro* under optimized conditions to obtain metaphase II (MII) oocytes. Quality of the oocytes from IVM and superovulation was tested by *in vitro* fertilization (IVF) and embryo development.

**Results:**

Less than ∼5 oocytes per animal could be isolated by superovulation only. However, we successfully obtained 20.3±2.9 oocytes per animal by IVM (16.0±2.5) and superovulation (4.3±1.3) in this study. Moreover, IVF and embryo development studies suggest that IVM oocytes have even better quality than that from superovulation The latter never developed to beyond 2-cell stage as usual while 9% of the former developed to 4-cells.

**Significance:**

We have successfully established the protocol for isolating oocytes from deer mice with high yield by IVM. Moreover, this is the first ever success to develop *in vitro* fertilized deer mice oocytes beyond the 2-cell stage *in vitro*. Therefore, this study is of significance to the use of deer mice for reproductive biology research.

## Introduction

The most widely used animal model systems for biomedical research are laboratory mice and the vast majority of the animals including the most widely used C57BL/6 (B6) strain fall into what are known as the classic inbred strains [1]. However, because of the unnatural, man-made history of the inbred strains, their haplotype structure and genetic make-up do not resemble that of humans or even wild mice [2], [3]. As a result, translation of the results obtained using the inbred laboratory mice to human (outbred) is limited.

The genuses *Peromyscus* (also called deer mice because their fur color resembles deer) are indigenous rodents in North American [4], [5]. *Peromyscus* have been used as the animal model in studies of phylogeography, seciation, chromosomes, and population genetics and evolution because they are not difficult to maintain in an animal facility and most importantly, they are probably more appropriate than inbred laboratory mice for research aimed for medical applications due to their outbred nature as with humans. They are also useful for aging research because they have a maximum life span of 5 to 7 years that is closer to human life span, compared to the 2 to 3 years of life span for *Mus musculus* [6], [7].

Although *Peromyscus* have been used as the model animals in a variety of research fields mentioned above, it is still in its infancy to use the animals for reproductive biology and assisted reproductive technologies (ARTs) for which a large supply of oocytes is of utmost importance [4], [8], [9]. PMSG (pregnant mare’s serum gonadotropin) and hCG (human chorionic gonadotropin) are the two hormones that have been used to retrieve oocytes from laboratory mice usually with high yield (more than 20 per animal) [10], [11], [12]. However, due to the large variation in response to hormone stimulation from species to species [12], [13] , the yield of oocyte retrieval from deer mice by superovulation using the two hormones has been dismal according to the literature to date: Less than ∼5 oocytes per animal could be obtained [5], [8]. Consequently, an optimized protocol for isolation of deer mice oocytes, its *in vitro* fertilization (IVF), and *in vitro* embryo culture and development has not been well established [4], [9].

Besides superovulation, another way to obtain oocytes is *in vitro* maturation (IVM) by culturing non-ovulated, immature oocytes in ovarian follicles *in vitro*. For example, a large quantity of high quality oocytes have been obtained by IVM of the immature oocytes in antral follicles for assisted reproduction of not only mice [12], [14], rats [15], porcine [16], [17], and bovine [18], [19] but also humans [20]. However, no IVM study for obtaining deer mice oocytes has been reported in the literature and we found that there were many antral follicles in the ovarian cortex of deer mice after they were treated with PMSG and hCG.

In this study, we employed IVM to improve the yield of oocyte isolation from deer mice close to that of most laboratory mice. We subsequently evaluated the development to 2- and 4-cell stages of the oocytes after *in vitro* fertilization (IVF) to determine the quality of the oocytes derived from IVM as compared to that from superovulation. Our results show that the IVM oocytes have even better quality than their counterparts from superovulation in terms of development. Therefore, this study is of significance to the use of deer mice as a more appropriate model than inbred laboratory mice for reproductive biology research.

## Materials and Methods

### Animals and Ethics Statement

*Peromyscus maniculatus bairdii* (BW stock) deer mice were purchased from the *Peromyscus* Genetic Stock Center at the University of South Carolina, Columbia, SC and were maintained on a 16∶8 h light-dark cycle. All animal use procedures were approved by the Institutional Animal Care and Use Committee (IACUC) at The Ohio State University (IACUC #: 2011A00000084) and all efforts were made to minimize animal suffering.

### Chemicals

L-15 Leibovitz-glutamax, fetal bovine serum (FBS), and M2 medium were purchased from Invitrogen, Hyclone, and millipore (USA), respectively. Unless specifically noted otherwise, all other chemicals were purchased from Sigma (USA).

### Isolation of Oocytes by Superovulation

Female deer mice of 12 to 14-week old were used for oocyte isolation study. The animals were hormone treated using two different methods. The first method involves the use of two administrations of PMSG according to a recent publication which reported that it could improve the yield of oocyte production by superovulation from deer mice [5]. This method is therefore called the 2-PMSG method hereafter. The second method has only one PMSG administration as has been commonly done for isolating oocytes from laboratory mice and hamster (another outbred species) [21] and is therefore called as the 1-PMSG method hereafter.

For the 2-PMSG method, the animals were first administered with PMSG of various doses (5, 7.5, and 15 IU) and a second administration of PMSG at the same dose of the 1^st^ administration was then performed after 24 h, followed by hCG administration at the same dose (5, 7.5, oand15 IU) of PMSG 48 h later to induce ovulation of oocytes from ovary. Although only 15 IU was studied in [5] for the 2-PMSG method, we also studied hormone doses at 5 and 7.5 IU for direct comparison with the 1-PMSG method for which the animals were injected with PMSG at two different doses (5 or 7.5 IU) and after 56 h, ovulation of oocytes was induced by administration of hCG at the same dose of PMSG (5 or 7.5 IU). All hormone administration was done by intraperitoneal (i.p.) injection. The animals were euthanized by cervical dislocation 18 and 15 h after hCG administration for the 2- and 1-PMSG methods, respectively. Of note, euthanizing the animal at a significantly later time (18 h as compared to the usual 15 h) after hCG administration was reported to increase the yield of oocyte retrieval by superovulation from deer mice using the 2-PMSG method [5]. Both ovaries and oviducts with an attached fragment of the uterus were then excised for further IVM and superovulation studies, respectively.

Superovulated cumulus–oocyte complexes (COCs) were retrieved by flushing the oviducts using M2 medium. To remove cumulus cells, the COCs were incubated in M2 medium containing 200 IU/ml hyaluronidase at 37°C for up to 3 min and further washed several times using fresh M2 medium.

### Isolation of COCs from Antral Follicles and IVM of the Follicular Oocytes

The ovaries retrieved from euthanized females injected with 5 IU PMSG and hCG using the 1-PMSG approach were placed in L-15 Leibovitz-glutamax medium supplemented with 10% (v/v) heat-inactivated FBS. COCs were isolated from antral follicles mechanically by carefully and gently puncturing the follicles using a pair of 30 gauge needles attached to disposable syringes under a stereomicroscope. The COCs containing immature oocytes in the antral follicles from both the outer (ovarian surface) and inner (deep ovarian tissue) layer of ovarian cortex were collected. IVM of oocytes in the COCs were done by culturing them for 17, 18, 19 or 20 h in 500 ml of minimum essential medium-α containing Earle’s salts (Invitrogen) and supplemented with 10 mg/ml streptomycin sulfate, 75 mg/ml penicillin G and 5% (v/v) heat-inactivated FBS covered with 250 µl of mineral oil in a 4-well plate at 37°C in 5% CO2 air [12]. After IVM, COCs were collected and incubated in M2 medium containing 200 IU/ml hyaluronidase at 37°C for up to 3 min to remove cumulus cells, and further washed twice in fresh M2 medium to obtain clean oocytes. Matured oocytes at the metaphase II stage were judged by the appearance of the first polar body.

### In Vitro Fertilization (IVF) and Embryo Culture

To obtain sperm for IVF of oocytes, male deer mice of 12 to 14-week old were euthanized by cervical dislocation and epididymides were collected by dissection. They were then placed in the central well of an IVF dish with 1 ml TYH medium, a modified Krebs-Ringer bicarbonate solution containing 5.56 mM glucose, 1.0 mM sodium pyruvate, 4****mg/ml bovine serum albumin (BSA), and antibiotics [9]. After making 5–7 longitudinal cuts using syringe needle on each epididymis, they were incubated for 20 min at 37°C in 5% CO_2_ air to allow for sperm dispersion. The sperm suspensions were incubated for 2 h at 37°C in 5% CO_2_ air for capacitation. For IVF, 5 metaphase II (MII) oocytes derived from either superovulation or IVM were inseminated with 2×10^4^ sperm in a 200 µl droplet of TYH medium for 4.5 h. Fertilized oocytes were subsequently cultured in a drop of 50 µl of Hoppe and Pitts medium modified by removing sodium lactate and increasing the sodium chloride concentration to 5.97 g/ml [22] at 37°C in 5% CO_2_ air. Development of the fertilized oocytes was monitored under a phase contrast light microscope (Nikon 80i) for the formation of 2-pronuclei, 2-cell, and 4-cell stages at various times for up to 7 days.

### Statistical Analysis

A generalized linear model (PROC-GLM) and one-way ANOVA in a Statistical Analysis System (SAS) program was employed for statistical analysis to determine the *p* value between various treatments. The differences were taken as significant when the *p* value was less than 0.05.

## Results and Discussion

### Isolation of Oocytes From Deer Mice by Superovulation

The results of superovulation using both the 1- and 2-PMSG methods and various hormone doses are given in Table 1. As shown in the table, the 1-PMSG method with 5 IU PMSG and hCG resulted in the most total number of oocytes (4.2±1.8) and highest percentage (71%) of mature MII oocytes. An increase of the hormone dose to 7.5 IU leads to a decrease in the percentage of MII oocytes to 35%. For the 2-PMSG method, a hormone dose of 5 IU gives the highest yield of oocyte production per animal while a dose of 7.5 IU gives the highest percentage of MII oocytes. The oocytes retrieval decreases with the further increase in hormone does to 15 IU. These observation is different from that reported in a recent study [5] where 15 IU of hormone was reported to result in the highest oocyte retrieval, which could be due to the difference in the source of the hormones used (Sigma for this study versus the National Hormone & Peptide Program at UCLA for the recent study). Nonetheless, the data for the 5 different superovulation protocols (i.e., 3 for the 2-PMSG method and 2 for the 1-PMSG method) in term of the percentage of MII oocytes and total number oocytes retrieved per animal are not significantly different with a *p* value of 0.0581 and 0.2226, respectively. The oocyte yield per animal using the various protocols are also not different from that reported in the recent study [5] where, however, no information on the percentage of MII oocytes per animal was given. Based on the data given in Table 1, further studies were performed using the 1-PMSG method with 5 IU PMSG and hCG because the 1-PMSG method is relatively simpler and a high concentration of hormones could have adverse effect on endocrine function and gametogenesis of ovaries [21], [23], [24], [25], cytoskeletal dynamics of oocytes, and embryogenesis [21], [26], [27].

**Table 1:** Comparison of oocyte retrieval by superovulation from deer mice using different methods and hormone doses (the total number of animals n = 5 for each condition): GVBD, germinal vesicle breakdown; MII, metaphase II; hCG**,** ***human chorionic gonadotropin; and PMSG,*** pregnant mare serum gonadotropin.

Method	Dose (IU)	Total # of oocytes retrieved	# (%)^a^ of oocytes at		# (in mean±SD) of oocytes retrieved per animal
			GVBD	MII	
**2-PMSG** b	5	19	19 (100)	7 (37)	3.8±0.8
	7.5	16	16 (100)	11 (69)	3.2±2.3
	15	10	10 (100)	5 (50)	2.0±1.6
**1-PMSG** c	5	21	21 (100)	15 (71)	4.2±1.8
	7.5	20	20 (100)	7 (35)	4.0±2.1

a Percentage of MII oocytes out of total oocytes retrieved.

b The 2-PMSG method includes a second administration of PMSG 24 h after the first one at the same dose, and hCG at the same dose was applied 48 h after the 2^nd^ PMSG administration. Oocytes were isolated from the oviduct 18 h after hCG.

c The 1-PMSG method consist one PMSG administration at the given dose and hCG at the same dose was applied 56 h later. Oocytes were isolated from the oviduct 15 h after hCG administration.

The low yield of oocytes retrieved by superovulation from deer mice could be a result of the varied response of ovarian tissue to exogenous hormones among different strains since superovulation does not change the level of endogenous gonadotropins [28], [29]. Another possible explanation is related to stresses that could decrease ovulation in mammals by stimulating the hypothalamus-pituitary-gonadal axis to inhibit endogenous gonadotropin secretion [12], [30], [31]. Deer mice might be more responsive to stresses in their environment as natural animals. In addition, the promiscuous nature of deer mice used in this study may influence their capability of ovulation as promiscuous *Mus spicilegus* were shown not to respond to standard superovulation treatment either [12], [31], [32]. Further studies are needed to identify the exact mechanism that is responsible for the low yield of oocyte superovulation from the deer mice used in this study.

### Isolation of Oocytes by IVM of Immature Oocytes in Antral Follicles

**Figure 1:**
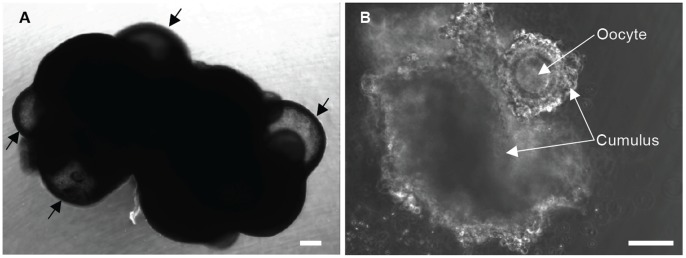
Morphology of deer mice ovary aftersuperovulation treatment and cumulus-oocyte complexes (COC). (A) A typical deer mice ovary collected after superovulation treatment showing many antral follicles (arrows) containing COC in the ovarian cortex. (B) A typical COC isolated from antral follicles by follicular puncture. Scale bar: 500 and 80 µm in (A) and (B), respectively.

As a result of the lack of response to superovulation, many antral follicles (arrows in Fig. 1A) were observed in the deer mouse ovary instead of being ovulated after superovulation treatment with PMSG and hCG. Moreover, we also found many inner antral follicles deep in the ovarian cortex after removing all the antral follicles on the surface. The presence of the inner antral follicles presumably is a result of the low efficiency of the surface antral follicles to ovulate and release oocytes from the ovary into oviduct. In other words, the inner antral follicles would become surface ones if those on the surface earlier could ovulate efficiently. The cumulus-oocyte complexes (COCs, Fig. 1B) contained in these antral follicles were mechanically isolated by follicular puncture and further cultured to obtain mature MII oocytes. As shown in Table 2, the number of COCs containing immature oocytes retrieved per animal from both the outer and inner antral follicles were significantly much more than that retrieved from only surface antral follicles (16.0±2.5 vs 5.3±2.8; *p*<0.0001). The total number of oocytes retrieved from superovulation and IVM using both the surface and inner antral follicles is 20.3±2.9, which is significantly (*p*<0.0001) much more than that obtained using only the surface antral follicles (9.3±3.8). Moreover, an IVM time of 19 h is significantly better than 17 (commonly used in the literature for laboratory mice) [12]), 18 or 20 h for the immature GVBD oocytes in the isolated COCs to further develop to the MII stage (58% for 19 h vs. 29, 35, and 21% for 17, 18, and 20 h, respectively; *p*<0.0001). The percentage of MII oocyte derived from IVM for 19 h is similar to that of superovulation (58 vs. 61%).

**Table 2:** Comparison of number of oocytes retrieved by superovulation and *in vitro* maturation (IVM) from deer mice: GVBD, germinal vesicle breakdown; COCs, cumulus-oocyte complexes; and MII, metaphase II.

# of deer mice	Superovulation			In vitro maturation (IVM)					Total
	Total # of (GVBD)oocytes (per animal)^a^	# of MIIoocytes	Total # (%)^b^of MII oocytes	Type of antralfollicles	Total # of COCs(per animal)^a^	IVM timein h	# of GVBD oocytes	# (%)^b^ of GVBDoocytes developedto MII Phase	# of oocytes retrieved (per animal)^a^
12	47 (3.9±0.9)	26	76 (61%)	Surface	64 (5.3±2.8)	17	64	12 (19)	111 (9.3±3.8)
18	77 (4.3±1.3)	50		Surface and inner	288 (16.0±2.5)	17	72	21 (29)	365 (20.3±2.9)
						18	72	25 (35)	
						19	72	42 (58)	
						20	72	15 (21)	

a The data for number of oocytes per animal is in mean ± SD.

b Percentage of MII oocytes out of total oocytes retrieved.

**Figure 2:**
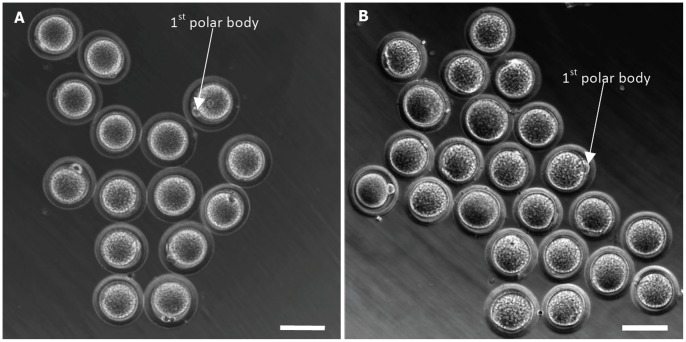
Morphology of deer mice oocytes isolated by superovulation and *in vitro* maturation (IVM). Typical phase contrast micrographs of oocytes derived by superovulation (A) and IVM (B, 19 h maturation) of antral follicles from deer mice hormone-primed using 5 IU PMSG and hCG using the 1-PMSG method. The first polar bodies are observable in MII oocytes in both panels. Scale bars: 70 µm.

In summary, a total of ∼20 oocytes per animal could be isolated using both superovulation and IVM of antral follicles and the number of oocytes retrieved from IVM is about 4 times of that from superovulation (∼16 vs. 4). Typical images of the MII and GVBD oocytes obtained by superovulation and IVM (19 h) of antral follicles are shown in Fig. 2A and B, respectively. The 1^st^ polar body is clearly visible in MII oocytes derived using both superovulation and IVM.

### *In vitro* Fertilization and Development of MII Oocytes Derived from Superovulation vs. IVM

**Figure 3:**
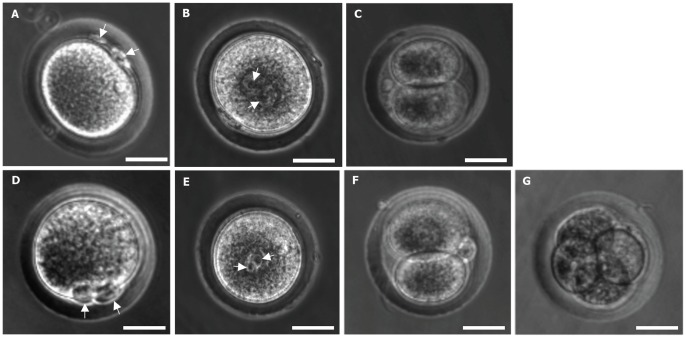
Morphology of *in vitro* fertilized (IVF) deer mice oocytes at various stages of development *in vitro*. Typical phase contrast micrographs of developing embryos derived from IVF of MII oocytes retrieved from superovulation (A-C) and IVM (D-G). Fertilized oocytes with first and secondary polar bodies are clearly visible as indicated by arrows in panels (A) and (D), which was followed by the appearance of two pronuclei (one from oocytes before fertilization and one from sperm) as indicated by arrows in panels (B) and (E) and further development to 2-cell (C and F), and 4-cell (G) stages *in vitro*. Scale bar: 30 µm.

To assess the quality of the MII oocytes isolated by superovulation vs. IVM, *in vitro* fertilization (IVF) were performed using 47 MII oocytes from each of the two groups. The results are given in Table 3 and Fig. 3. Successful IVF of the MII oocytes was first judged by the appearance of secondary polar body as illustrated in Fig. 3A and D and then the appearance of two pronuclei (one from oocytes and the other from sperm) as shown in Fig. 3B and E. The percentages of successful IVF of the MII oocytes from both groups were perfect (100%, Table 3) when judged by the appearance of two polar bodies while it was 87% judged by the appearance of two pronuclei. Further development of the fertilized oocytes to 2-cell stage and beyond (if any) was monitored and the data are given in Table 3 with representative images of the embryos at various stages being shown in Fig. 3C and F. The percentage (23%) of the fertilized oocytes from IVM developed to the 2-cell stage is higher (but not significantly, *p* = 0.45) than that from superovulation (17%). A total of 4 (∼9%) of the fertilized oocytes from IVM developed to 4-cell stage (Fig. 3G). However, none of the fertilized oocytes from superovulation developed to beyond the 2-cell stage, which is significantly (*p* = 0.04) lower than that of the fertilized IVM oocytes. These results suggest that the IVM oocyte could have better quality than that from superovulation. Nonetheless, recent studies show that *in vitro* maturation could induce epigenetic alterations [33], [34], [35]. Therefore, the superiority in quality of IVM oocytes as compared to that of superovulation could not be ascertained until viable and normal animals are produced from the IVM oocytes.

**Table 3:** Development of embryos derived from IVF (*in vitro* fertilization) of metaphase II (MII) deer mice oocytes obtained by superovulation vs. IVM (*in vitro* maturation): The total number of animals used n = 23.

Type	# of MII oocytes	# of fertilized oocytes (%)^a^	# (%)^b^ of embryos developed to		
			2-pronuclei	2-cell	4-cell
**Superovulation**	47	47 (100%)	13^c^ (87)	8 (17)	0 (0)
**IVM**	47	47 (100%)	13^c^ (87)	11 (23)	4 (9)

a Fertilized oocytes were judged by the appearance of secondary polar body as shown in Fig. 3A and D.

b Percentage of fertilized oocytes.

c A total of 15 fertilized oocytes was monitored for the formation of two pronuclei.

Although the focus of this work is on improving the efficiency of oocyte isolation by IVM of COCs, it is worthy to note that the above data from our study should represent a significant step forward in assisted reproduction of deer mice because the highest developmental stage that could be achieved *in vitro* before our study for deer mice oocytes after *in vitro* fertilization is 2-cell at a rate of only 2% [9]. Nevertheless, the percentages of IVF oocytes developed to 2-cell (23%) and 4-cell (9%) stages are still low, suggesting that more research needs to be done to completely overcome the developmental block at 2-cell stage. Moreover, it was reported that superovulated 2-cell embryo from deer mice could not develop to beyond 8-cell stage *in vitro* and only superovulated 8- to 16-cell stage embryos could be developed to blastocysts [5], suggesting another developmental block at the 8-cell stage. Therefore, the challenge is expected to be tremendous to overcome the potential developmental blocks so that *in vitro* fertilized oocytes could be developed to the blastocyst stage. Research is ongoing in our lab with the hope to resolve the challenge by utilizing novel biomaterials and identifying new chemokines to optimize the culture condition of the IVF oocytes so that they can develop to blastocysts that can be further implanted to produce viable and normal animals.

### Summary

In this study, we report the method for isolation of a large number of oocytes (∼20) per animal from deer mice by *in vitro* maturation of immature oocytes in antral follicles in both the surface and inner layer of ovarian cortex after PMSG and hCG treatment, in addition to superovulation. MII oocytes could be obtained with high efficiency (∼60%) from the oocytes in antral follicles with an optimized IVM time of 19 h. Moreover, the *in vitro* fertilized oocytes could further develop to 2-cell and for the first time, the 4-cell stage. Further studies are needed to optimize the embryo culture condition so that the *in vitro* fertilized oocytes could develop to 8-cell and blastocyst and eventually be used to produce viable and normal animals.
